# Curcumin, EGCG and apigenin in cervical cancer: mechanistic insights and therapeutic potential

**DOI:** 10.3389/fphar.2025.1592395

**Published:** 2025-07-01

**Authors:** Abdulaziz Asiri, Bayan T. Bokahri, Sadaf, Alaa Abdulaziz Eisa, Hashim M. Aljohani, Wesam Nofal, Tahreem Kausar, Mohammad Zeeshan Najm

**Affiliations:** ^1^ Department of Medical Laboratory Sciences, College of Applied Medical Sciences, University of Bisha, Bisha, Saudi Arabia; ^2^ Department of Clinical Laboratory Sciences, Faculty of Applied Medical Sciences, Umm Al-Qura University, Makkah, Saudi Arabia; ^3^ Department of Biotechnology, Jamia Millia Islamia, New Delhi, India; ^4^ Department of Clinical Laboratory Sciences, College of Applied Medical Sciences, Taibah University, Medina, Saudi Arabia; ^5^ Department of Pathology and Laboratory Medicine, College of Medicine, University of Cincinnati, Cincinnati, OH, United States; ^6^ Department of Medical Laboratory Technology, Faculty of Applied Medical Sciences, Northern Border University, Arar, Saudi Arabia; ^7^ Department of Food and Nutrition, Institute of Home Economics, Delhi University, New Delhi, India; ^8^ School of Biosciences, Apeejay Stya University, Gurgaon, India

**Keywords:** HPV, cervical cancer, curcumin, EGCG, apigenin

## Abstract

Cervical cancer (CC) continues to be the major cause of death from cancer in women worldwide and highlights the necessity for novel therapeutic approaches that target key oncogenic pathways. Conventional treatments, including chemotherapy and radiotherapy, exhibit significant limitations, including drug resistance, toxicity, and limited durability of response, highlighting the need for improved strategies. In recent years, Phytochemicals like curcumin, EGCG, and apigenin have demonstrated potent anticancer effects by modulating multiple dysregulated pathways in CC. These natural compounds exhibit multi-targeted effects, influencing signaling cascades such as PI3K/Akt, JAK/STAT, Wnt/β-catenin, and NF-κB, which drive tumor progression and metastasis. While plant-derived compounds like curcumin, EGCG, and apigenin have shown promising anticancer effects in preclinical models, there is a critical lack of comprehensive reviews that integrate mechanistic insights, clinical progress, and bioavailability challenges. Specifically, gaps remain in correlating these compounds’ modulation of cervical cancer-relevant signaling pathways with clinical outcomes, as well as in synthesizing recent innovations in nanotechnology that enhance their pharmacokinetics. A comparative evaluation highlights their mechanistic overlaps in regulating oncogenic signaling and their potential for synergistic combinations with conventional therapies to enhance treatment efficacy and overcome drug resistance. While bioavailability and systemic stability hinder clinical translation, advancements in nanotechnology and targeted delivery systems offer promising solutions. Future research should prioritize optimizing formulations and conducting large-scale clinical studies to facilitate the integration of plant bioactives into CC therapy, ultimately improving patient outcomes. Despite increasing interest in phytochemicals for cervical cancer treatment, current literature lacks comprehensive synthesis of studies addressing their molecular mechanisms, clinical efficacy, and novel strategies such as nanotechnology to enhance bioavailability.

## Graphical Abstract

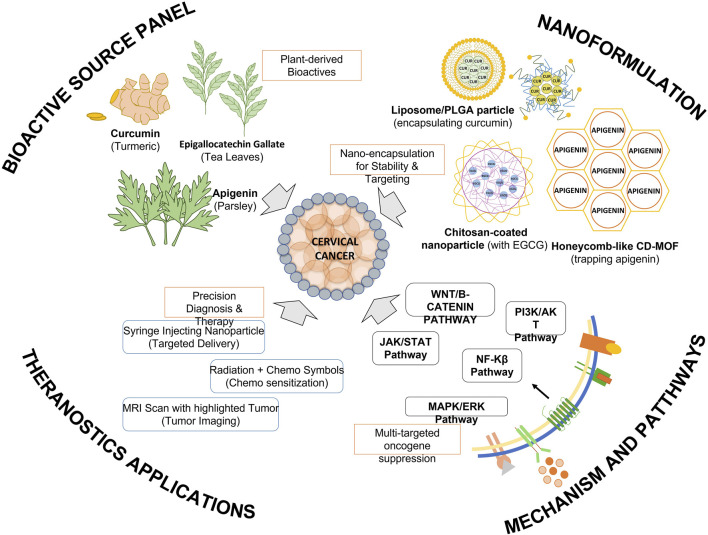

## 1 Introduction

Cervical cancer (CC) remains a major public health challenge, with nearly 20 million new cancer diagnoses and 9.7 million deaths reported worldwide in 2022 (Bray Bsc et al., 2024). Globally, CC ranks as the fourth most diagnosed malignancy among women, posing a significant threat, especially in low-resource countries with limited access to preventive measures. High-risk human papillomavirus (HPV) is the primary cause of almost all CC cases, particularly those with persistent infection (World Health Organization, 2024). Additional risk factors such as HIV infection, prolonged contraceptive use, and high-risk sexual behaviors also influence CC susceptibility and progression (Nagelhout et al., 2021; Zhao et al., 2022). Despite the availability of effective HPV vaccines and widespread screening programs, barriers such as vaccine accessibility and uptake hinder comprehensive prevention efforts. Standard treatments like chemotherapy and radiotherapy remain common but are often associated with significant side effects, therapy resistance, and recurrence (Burmeister et al., 2022). To improve therapeutic outcomes, it is essential to understand the molecular drivers of CC progression and resistance. A growing body of research has identified several dysregulated oncogenic signaling pathways—PI3K/AKT (Peng et al., 2022), NF-κB (Deng et al., 2024), MAPK/ERK (Braicu et al., 2019), Wnt/β-catenin (Sharma et al., 2021), and JAK/STAT (Valle-Mendiola et al., 2023). These pathways regulate critical cellular processes such as apoptosis, proliferation, angiogenesis, immune evasion, and metastasis. Their central role in tumor growth and therapy resistance makes them promising targets for novel therapeutic approaches, including plant-derived bioactive compounds. Valued for their low toxicity, broad-spectrum efficacy, and cost-effectiveness, phytochemicals like curcumin, apigenin, and EGCG have demonstrated potent anticancer effects by modulating multiple dysregulated pathways in CC. For example, curcumin inhibits PI3K/AKT and NF-κB signaling, while apigenin and quercetin influence MAPK/ERK and JAK/STAT pathways, respectively (Braicu et al., 2019; Deng et al., 2024; Peng et al., 2022; Valle-Mendiola et al., 2023). We conducted a narrative review using databases including PubMed, Scopus, and Web of Science, focusing on studies published between 2010 and 2025. Keywords included “cervical cancer,” “phytochemicals,” “curcumin,” “EGCG,” “apigenin,” and “nanotechnology.” Studies were selected based on relevance to cervical cancer, mechanistic insight, therapeutic potential, or clinical and preclinical outcomes.

Natural compounds have a long-standing role in cancer therapy, with scientific exploration accelerating in the mid-20th century through large-scale screening programs (Chandra, 2012; Newman and products, 2016). This growing interest highlights the therapeutic potential of plant-derived agents in modern cancer care. Phytochemicals such as curcumin (Zhang et al., 2023), epigallocatechin gallate (EGCG) (Hussain and Ashafaq, 2018), and apigenin (Naponelli et al., 2024) have shown promising anticancer activity in preclinical studies. Curcumin induces p53-mediated apoptosis in cervical cancer (CC), addressing the resistance often seen with conventional therapies (Zhang et al., 2023). EGCG counters the aberrant activation of the PI3K/Akt/mTOR pathway, a known contributor to chemoresistance, thereby potentially sensitizing tumors to chemotherapeutic agents (Y. Q. Wang et al., 2018). Similarly, apigenin targets the PI3K/Akt and integrin β1-FAK signaling pathways, disrupting tumor survival mechanisms and limiting growth, particularly in treatment-resistant cancers (Tripathi et al., 2024). However, their clinical application is limited by poor bioavailability and metabolic instability, which advances in drug delivery and nanotechnology aim to overcome to enhance their therapeutic efficacy. This review integrates recent preclinical and clinical research on curcumin, EGCG, and apigenin in CC, highlighting their ability to disrupt essential oncogenic pathways, synergize with conventional therapies, and target cancer stem cells. By addressing bioavailability and translational challenges, this review aims to support the development of more effective and targeted treatment strategies.

## 2 Overview of cervical cancer

Cervical cancer (CC), originating in the cervix of the uterus, ranks as the fourth most common cancer among women globally, with 1,413,316 new cases reported in 2022 (World Health Organization, 2025). Its incidence and mortality vary across regions, with low-income countries—often lacking sufficient healthcare infrastructure—accounting for 80%–90% of global cases (Hull et al., 2020). In India alone, there were 79,906 deaths attributed to CC in 2022 (*Cancer Today*, 2022). Persistent infection with high-risk human papillomavirus (HPV) is the principal cause of CC, responsible for 99% of cases (Okunade, 2020). However, HPV infection alone is not sufficient to drive disease progression. Contributing factors include a weakened immune system, prolonged oral contraceptive use, smoking, early sexual activity, multiple sexual partners, and co-infections with other sexually transmitted diseases (Mohanty and Ghosh, 2015). HPV, a double-stranded DNA virus from the Papillomaviridae family, includes over 200 genotypes. High-risk types such as HPV16 and HPV18 account for 70%–90% of CC cases worldwide, with HPV16 alone responsible for nearly 90% of cases in India (Ahmed et al., 2017; Muñoz et al., 2003).

Treatment approaches vary by disease stage and include surgery, chemotherapy, radiation, or a combination of these modalities (Burmeister et al., 2022). Early-stage CC is commonly treated with surgical interventions, which may be effective in early-stage disease but can lead to complications such as infertility, sexual dysfunction, and lymphedema, impacting the patient’s quality of life (Greggi et al., 2020). Chemotherapeutic agents like cisplatin, although widely used, often cause systemic toxicity, including nephrotoxicity, ototoxicity, myelosuppression, and gastrointestinal distress (Elmorsy et al., 2024). Moreover, the development of chemoresistance, particularly to cisplatin, poses a major challenge, reducing long-term treatment efficacy (Nguyen et al., 2022). Radiotherapy, while crucial for locally advanced disease, is not without adverse effects, including radiation-induced proctitis, cystitis, and premature ovarian failure (Chargari et al., 2022). Despite their effectiveness, conventional therapies frequently result in severe side effects and significant recurrence rates (Tripathi et al., 2024). These challenges have prompted the exploration of alternative therapies, including immunotherapy (Naumann and Leath, 2020), targeted treatments (Mutlu et al., 2022), and genome-editing technologies such as CRISPR/Cas9 (Inturi and Jemth, 2021) and RNA interference (Jung et al., 2015). Although many of these remain experimental and costly.

### 2.1 Role of HPV infection in cervical cancer progression

Human papillomaviruses (HPV) belong to the Papillomaviridae family and are small, double-stranded DNA viruses, with over 200 identified types. Among these, high-risk HPV strains—particularly HPV-16 and HPV-18—are strongly associated with cervical cancer, contributing to over 70% of global cases (*HPV and Cancer -* NCI, 2025). Although sexual contact remains the primary route of transmission and most infections are cleared by the immune system, persistent infections with oncogenic HPV types can lead to malignant transformation in cervical epithelial cells (R. Wang et al., 2020). Cervical carcinogenesis involves a complex series of molecular events including viral genome integration, immune evasion, and host cell cycle dysregulation, ultimately leading to cervical intraepithelial neoplasia (CIN) and invasive cervical cancer (Balasubramaniam et al., 2019).

HPV infection initiates when the virus gains access to the basal epithelial cells of the cervix through microabrasions in the mucosal lining (Stanley, 2012). Unlike many viruses, HPV remains localized to the stratified squamous epithelium and does not enter the bloodstream, enabling it to efficiently evade immune surveillance (Hewavisenti et al., 2023). Within the basal cells, the virus establishes a latent infection. As these cells differentiate and migrate toward the epithelial surface, viral replication begins, producing new virions (McBride, 2008). The early proteins E1 and E2 are crucial for viral DNA replication and maintenance of genome integrity. As the infection progresses, additional oncoproteins—E5, E6, and E7—intervene in host cellular pathways to facilitate viral persistence and oncogenic transformation (Porter and Marra, 2022). HPV infection primarily affects the transformation zone of the cervix, leading to dysplastic changes clinically classified as CIN 1, 2, or 3, reflecting increasing levels of cellular abnormality (Siebers et al., 2018). While most HPV infections resolve within a year, persistent infections in 10%–20% of cases can progress to cancer (Shanmugasundaram and You, 2017). CIN1 involves mild changes in the lower third of the cervical lining, whereas CIN2 and CIN3 represent more extensive epithelial abnormalities (Matsumoto et al., 2011). Progression involves inflammation-induced reactive oxygen species (ROS), which generate DNA breaks that facilitate viral genome integration into host DNA. This process activates HPV oncoproteins E6 and E7, which are crucial in sustaining the malignant phenotype (Williams et al., 2011). E6 promotes the degradation of the tumor suppressor p53, while E7 interferes with the retinoblastoma protein (Kennedy et al., 2014), collectively contributing to the development of invasive CC.

**FIGURE 1:**
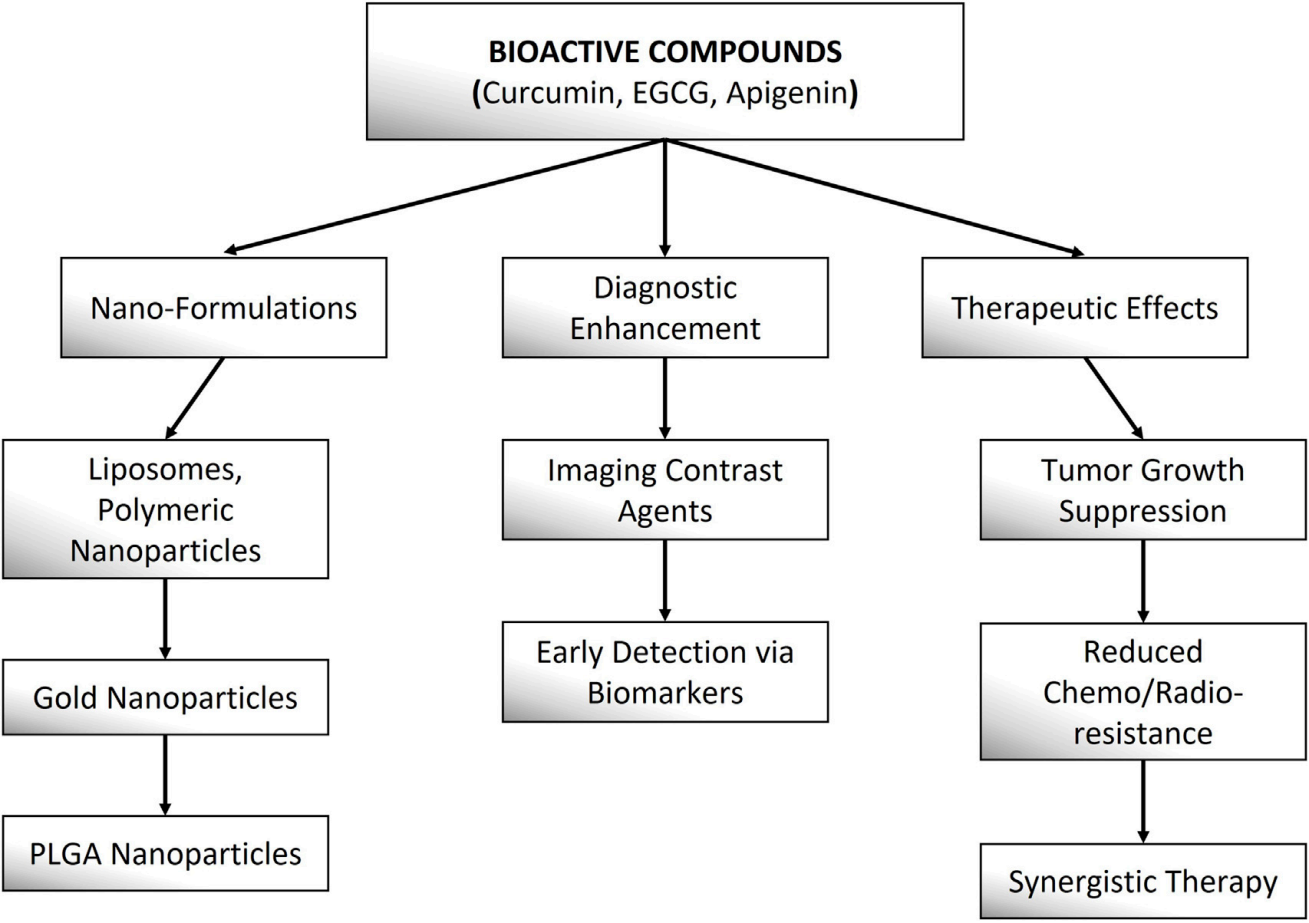
The progression of HPV-driven cervical cancer, illustrating key stages of infection, viral genome integration, and oncogenic transformation.

Among the viral oncogenes, E6 and E7 are particularly significant in the development of cervical cancer. The E6 protein targets the tumor suppressor p53 by promoting its degradation via the ubiquitin-proteasome system. This prevents p53 from initiating apoptosis in response to DNA damage, thereby allowing genetically unstable cells to survive and proliferate (Tomaić, 2016). Meanwhile, the E7 protein disrupts the cell cycle by binding to and inactivating the retinoblastoma protein (pRb), a key regulator of E2F transcription factors (Jansma et al., 2014). This interaction results in uncontrolled cell division, genomic instability, and continued proliferation of HPV-infected cells (Kassab et al., 2023). The E5 protein further contributes to carcinogenesis by promoting cell growth and reducing the expression of MHC molecules, impairing antigen presentation and weakening the host immune response (Figure 1). A hallmark of persistent HPV infection is its capacity to evade host immune surveillance. HPV-infected cells suppress interferon (IFN) responses and impair antigen presentation by dendritic cells, thereby reducing T-cell activation (Della Fera et al., 2021). Additionally, these infected cells secrete immunosuppressive cytokines such as IL-10 and TGF-β1, which help create a tumor-promoting microenvironment (Torres-Poveda et al., 2014). These immune evasion strategies allow HPV to persist within the cervical epithelium for extended periods, significantly increasing the risk of malignant progression. Despite advances in prophylactic vaccination with Gardasil and Cervarix—both of which offer highly effective protection against high-risk HPV subtypes 16 and 18 (Pathak et al., 2022)—cervical cancer continues to pose a serious health burden. This is especially true in low-resource countries, where limited access to vaccination and screening contributes to persistently high mortality rates (Hull et al., 2020).

### 2.2 Oncogenic signalling pathways involved in cervical cancer

Signaling pathways are intricate networks that convert extracellular information into specific cellular actions, such as proliferation, differentiation, or apoptosis. These pathways work according to the binding of extracellular stimuli with cell surface receptors, triggering cascades that amplify the initial signal and culminate in gene expression modifications. Disruptions in these signaling pathways are major contributors to various diseases, including diabetes, heart disease, autoimmune disorders, and cancer. In the context of cervical cancer (CC), high-risk human papillomaviruses (HPVs) utilize mechanisms to evade the host’s immune response along with host DNA integrity, primarily through their oncoproteins E6 and E7 (Lo Cigno et al., 2024). These proteins are always expressed in CC tumors and play a pivotal role in the growth and survival of HPV-related cancers.

**FIGURE 2:**
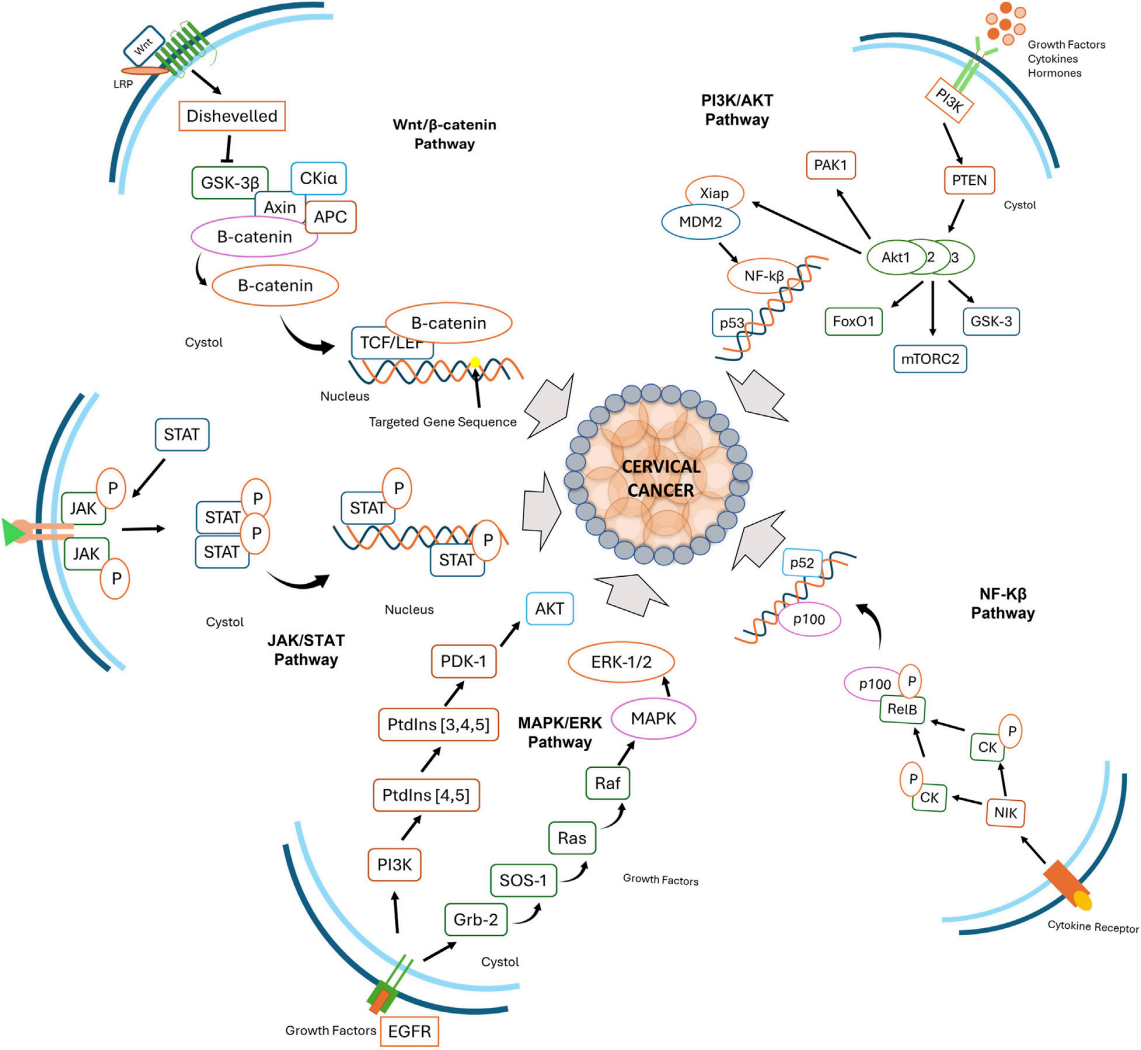
Key signaling pathways involved in cervical cancer progression, including Wnt/β-catenin, PI3K/AKT, JAK/STAT, MAPK/ERK, and NF-κB. These pathways regulate cellular proliferation, survival, immune evasion, and inflammation, contributing to tumor development and progression.

The progression of CC is governed by the dysregulation of multiple oncogenic signaling pathways, which promote uncontrolled proliferation, immune evasion, and metastasis (Wang et al., 2024). Comprehending these pathways is fundamental to designing targeted therapies and achieving better patient results. Various signaling pathways play a central role in CC, including PI3K/Akt (Peng et al., 2022), Wnt/β-catenin (Sharma et al., 2021), ERK/MAPK (Braicu et al., 2019), NF-κB (Deng et al., 2024), and JAK/STAT (Valle-Mendiola et al., 2023) (Figure 2). These pathways play a role in key aspects of tumor growth, including cell survival, invasion of surrounding tissues, and resistance to programmed cell death (apoptosis).

Stimulation of receptor tyrosine kinases (RTKs) by growth factors activates phosphatidylinositol 3-kinase (PI3K), which converts phosphatidylinositol-4,5-bisphosphate (PIP2) to phosphatidylinositol-3,4,5-triphosphate (PIP3), facilitating Akt activation. PTEN counteracts this by dephosphorylating PIP3. In the absence of PTEN, Akt activates mTOR, forming mTORC1 and mTORC2. mTOR activation leads to phosphorylation of downstream effectors such as S6K1 and 4E-BP1, enhancing translation and promoting cell proliferation, angiogenesis, and survival (Avan et al., 2016). In HPV-induced CCs, PIK3CA mutations and amplifications result in aberrant PI3K activation, enhancing epidermal growth factor receptor (EGFR) signaling and activating the MAPK/ERK pathway, disrupting cell cycle regulation (Zhang et al., 2015). PI3K also promotes viral RNA splicing via ASF/SF2, supporting viral persistence and oncogenesis. HPV-positive CCs often exhibit frequent Akt phosphorylation (p-Akt, Ser473), correlating with tumor progression and poor prognosis. Oncogenic PIK3CA mutations drive constitutive Akt activation, enhancing HPV-driven tumorigenesis. Akt suppresses E2F1’s proapoptotic function and promotes TopBP1 oligomerization to aid cell survival (Zhang et al., 2015). The mammalian target of rapamycin (mTOR) integrates signals for growth factors, nutrient availability, and energy metabolism. Upon HPV infection, mTOR activation supports viral replication and tumor growth. Over 60% of HPV-induced cancers show mTOR phosphorylation (p-mTOR, Ser2448), correlating with increased tumor aggressiveness and metastasis (Molinolo et al., 2012). mTOR enhances protein translation (via 4E-BP1, S6K) and suppresses autophagy, promoting cancer cell proliferation (Meric-Bernstam and Gonzalez-Angulo, 2009). Preclinical studies suggest that mTOR inhibitors (e.g., rapamycin, RAD001), combined with standard therapies, reduce tumor burden and prolong survival in HPV-associated cancers (Molinolo et al., 2012).

Downregulation of the Wnt/β-catenin signaling pathway is frequently observed in cervical cancer (CC) due to mutations, epigenetic modifications, and altered expression of pathway components. Specifically, Wnt7A is often downregulated, while Wnt10B, Wnt14, Wnt5A, Wnt11, Wnt4, and Wnt8A are overexpressed, all contributing to cancer progression (Yang et al., 2018). This pathway regulates essential cellular processes such as proliferation, adhesion, and differentiation (Liu et al., 2022). The Wnt pathway involves 19 ligands, 11 Frizzled (Fzd) receptors, and co-receptors LRP5/6, which can activate either the β-catenin-dependent (canonical) or β-catenin-independent (non-canonical) pathways (MacDonald and He, 2012). In the canonical pathway, Wnt ligands bind to Fzd receptors and LRP5/6, leading to the inhibition of GSK3β, which normally promotes β-catenin degradation. When Wnt signaling is active, β-catenin accumulates, translocates to the nucleus, and activates target genes that promote cancer, a feature characteristic of HPV-positive CC (Song et al., 2024). On the other hand, In the non-canonical pathways, such as the planar cell polarity (PCP) and Wnt/Ca^2+^ pathways, Wnt ligands like Wnt4 and Wnt11 bind Fzd receptors in the absence of LRP5/6, triggering intracellular cascades involving small GTPases (RhoA, Rac), JNK signaling, and calcium-dependent enzymes, regulating cell migration, polarity, and cytoskeletal organization (Pataki et al., 2015). Wnt signaling is also regulated by a group of antagonists, including Wnt inhibitory factor 1 (WIF1), Dickkopf (Dkk) proteins, and secreted frizzled-related proteins (sFRPs), which inhibit both canonical and non-canonical pathways. In CC, this regulation is often disrupted, with Dvl-1 overexpression and PP2A downregulation contributing to enhanced Wnt signaling (Yang et al., 2018). Additionally, epigenetic silencing of key regulators such as Axin, APC, and Wnt inhibitors like DKK-1, WIF1, and SFRP further disrupts Wnt pathway regulation, facilitating CC progression.

The JAK/STAT signaling pathway, critical for immune responses, apoptosis, migration, differentiation, and cell proliferation, is activated by cytokine and growth factor receptors that dimerize and activate JAK kinases. These kinases phosphorylate STAT proteins (STAT1-6), facilitating their nuclear translocation and transcriptional regulation of target genes (X. Hu et al., 2021). HPV oncoproteins, particularly E6 and E7, suppress this pathway to evade immune surveillance and promote persistent infection. HPV E6 inhibits Tyk2 phosphorylation, blocking STAT1/2 activation, while E7 interferes with STAT1/STAT2 heterodimerization, further suppressing IFN responses (Qi et al., 2024).

STAT1 expression follows a biphasic pattern in cervical carcinogenesis, being overexpressed in CIN1/2 but reduced in CIN3 and carcinoma *in situ* before rising again in invasive CC (Wu et al., 2020). HPV suppresses STAT1 expression and nuclear translocation, facilitating viral genome replication and immune evasion (Hong et al., 2011). STAT3 activation is strongly linked to CC malignancy, with HPV-positive cells showing increased STAT3 phosphorylation and autocrine IL-6 production, further enhancing STAT3 activation and modulating HPV gene expression (Haręża et al., 2022; Morgan and Macdonald, 2020). Additionally, STAT5 is upregulated in cervical tumors, correlating with disease severity, particularly in HPV16+ and HPV18+ CC cell lines (Morgan and Macdonald, 2020). While studies on STAT4 and STAT6 are limited, both have been implicated in immune regulation and tumor progression, with STAT4 overexpressed in squamous cell carcinoma and cervical adenocarcinoma, especially with lymph node metastasis (Gutiérrez-Hoya and Soto-Cruz, 2020). Further research is required to clarify the roles of STAT4 and STAT6 in CC progression and immune evasion.

The ERK (Extracellular signal-regulated kinase) and MAPK (Mitogen-Activated Protein Kinase) signaling pathway begins with the activation of Receptor Tyrosine Kinases (RTKs) utilizing growth factors (Almeida et al., 2004). Upon activation, RTKs stimulate Ras (Rat Sarcoma protein) and activate Raf protein (MAPKKK), MEK1/2 protein (MAPKK), and ultimately MAPK (e.g., ERK1/2) (Bahar et al., 2023). ERK1/2 phosphorylates numerous transcription factors, chromatin remodelers, and cytoskeletal proteins, impacting cell fate decisions (Zou et al., 2018). Other major MAPK pathways include p38 MAPK and JNK (c-Jun N-terminal Kinase), which regulate stress responses, inflammation, and apoptosis (Yue and López, 2020). The PI3K/AKT pathway closely interacts with MAPK/ERK signaling, as PI3K can be activated by Ras and RTKs, contributing to tumor progression and therapy resistance. Aberrant activation of the ERK/MAPK signaling cascade is a frequent observation in CC, attributable to the oncogenic activity of human papillomavirus (HPV) oncoproteins, growth factor receptor overexpression, and oncogenic genetic mutations. HPV E6 and E7 oncoproteins enhance ERK/MAPK activity, modulating key processes like invasion, migration, and anchorage-independent growth. Additionally, oncogenic E5 protein stabilizes VEGF expression through ERK activation, promoting angiogenesis and tumor growth. HPV-infected cells evade apoptosis and autophagy via MAPK-ERK signaling, contributing to therapy resistance and tumor persistence (Bonab et al., 2021).

The NF-κB signaling pathway plays a central role in immune regulation, inflammation, viral replication, and oncogenesis. It comprises five members: RelA (p65), RelB, c-Rel, NF-κB1 (p105/p50), and NF-κB2 (p100/p52) (Guo et al., 2024). NF-κB1 and NF-κB2 serve as precursors and are processed into their active forms—p50 and p52—via proteolytic cleavage (Ghosh and Wang, 2021). These proteins form homo- or heterodimers, which undergo post-translational modifications that enable their activation, nuclear translocation, and transcriptional regulation of target genes (Huang et al., 2010).

NF-κB activation occurs through two primary pathways. The canonical pathway is rapidly induced by cytokines (TNF-α, IL-1), microbial components, and Toll-like receptors, activating the IKK complex (IKK1, IKK2, NEMO). This leads to phosphorylation and degradation of IκB, releasing p50/p65 dimers that translocate into the nucleus to promote genes involved in inflammation, proliferation, and apoptosis resistance (Yu et al., 2020). In contrast, the non-canonical pathway is activated by receptors such as BAFFR, CD40, and RANKL. This route involves NIK-mediated activation of IKK1 and subsequent processing of p100 into p52, influencing immune development and cell survival (Sun, 2017).

HPV strategically exploits NF-κB signaling to support immune evasion and tumor progression. The viral E6 protein inhibits p65 transcriptional activity, downregulating immune gene expression and promoting viral persistence. Meanwhile, E7 disrupts the IKK complex, hindering NF-κB nuclear translocation and blunting host inflammatory responses (Lo Cigno et al., 2024). These manipulations allow HPV to evade detection during early infection and contribute to chronic inflammation and cervical carcinogenesis during later stages (Vats et al., 2021).

## 3 Therapeutic potential of bioactive compounds in cervical cancer

Over the past decade, researchers have extensively explored plant-derived bioactive compounds for their anticancer properties, particularly focusing on their ability to modulate key cellular pathways such as apoptosis, cell cycle regulation, angiogenesis, and metastasis. Recent studies have highlighted the potential of these compounds in cervical cancer therapy, demonstrating cytotoxicity against cancer cell lines and the ability to enhance chemosensitivity or overcome drug resistance (Silva-Pinto et al., 2025). *In vivo* and *in vitro* experimental studies highlight the potential of various phytochemicals used as a therapeutic intervention, particularly in cancer treatment (Lekhak and Bhattarai, 2024). Natural bioactive compounds have gained significant attention for their capability to target multiple pathways involved in CC, such as apoptosis induction, angiogenesis inhibition, and immune modulation (Asma et al., 2022). Several studies have identified natural products capable of suppressing CC progression through these mechanisms, offering promising prospects for developing novel, less toxic therapeutic approaches (Naponelli et al., 2024; Ratheesh et al., 2021; Wang et al., 2018).

Among the numerous plant-derived bioactive compounds explored for cervical cancer therapy, curcumin has emerged as one of the most extensively studied due to its multi-targeted mechanisms of action, favorable safety profile, and ability to modulate several key pathways implicated in cancer progression. Its broad-spectrum anticancer activity, supported by compelling *in vitro* and *in vivo* evidence, makes curcumin a prototypical compound for exploring phytochemical-based cervical cancer treatments. The following section provides a comprehensive overview of curcumin’s therapeutic potential in CC, including its molecular mechanisms, limitations, and recent advances in formulation strategies.

### 3.1 Curcumin

Curcumin, a polyphenolic phytochemical constituent of Curcuma longa rhizomes, has garnered substantial scientific interest owing to its pleiotropic pharmacological activities, encompassing anti-inflammatory, antioxidant, antibacterial, and antineoplastic effects (Zhang et al., 2023). Its chemical structure comprises two ortho-methylated phenols and a β-diketone moiety, contributing to its biological presence. With a molecular formula and molecular weight of C_21_H_20_O_6_, 368.37 g/mol, respectively, curcumin is characterized as an orange-yellow crystalline powder. However, its poor aqueous solubility and limited systemic bioavailability remain significant obstacles to its clinical translation (*Curcumin |* NLM, 2025b; Sharifi-Rad et al., 2020).

Initially recognized for its anticancer potential in 1985 by (Kuttan et al., 1985), curcumin has since been extensively studied for its effects on various cancers, including CC. The antineoplastic effects of curcumin in CC are predominantly mediated through the modulation of key oncogenic signaling cascades, including the PI3K/Akt, Wnt/β-catenin, and nuclear factor kappa β (NF/kβ) pathways. Curcumin elicits apoptosis in CC cell lines, such as HeLa, by generating reactive oxygen species (ROS), DNA damage induction, and chromatin condensation (Ghasemi et al., 2019). For instance, studies have demonstrated that curcumin enhances the expression of DNA damage and repair proteins, such as p-ATM, BRCA1, p-ATR, MDC1, DNA-PK, and MGMT, while also promoting the nuclear translocation of p-H2A.XSer140 and p-p53 (Zhang et al., 2023). Additionally, curcumin has been found to reduce COX-2 expression while increasing iNOS levels, leading to telomerase activity inhibition and key oncogenic pathways such as Ras and ERK, ultimately resulting in mitochondrial-mediated apoptosis (Ismail et al., 2019). Furthermore, curcumin-induced apoptosis is mediated through endoplasmic reticulum (ER) stress, activating key proteins like PERK, IRE-1α, and ATF6 (Zhang et al., 2018).

Curcumin also suppresses the proliferation of tumor cells by interfering with cell cycle progression. It induces apoptosis in a subset of G1-phase CC cells and G2/M cell cycle arrest by targeting the Wnt/β-catenin and NF-κB pathways (Hu et al., 2017). Research has shown that curcumin effectively arrests HeLa cells in the G1/S phase, triggering apoptosis via ROS-mediated mitochondrial damage, with increased caspase-8 enzyme and Bax expression after 48 h of treatment (Ratheesh et al., 2021). Another study demonstrated that curcumin upregulates tumor suppressor proteins such as p53 while inhibiting histone deacetylases (HDACs), cyclin-dependent kinases (CDKs), and cell cycle regulators like p21 and p27, further contributing to its antiproliferative effects (Talib et al., 2018).

Beyond controlling cell growth, curcumin hinders tumor spread by inhibiting metastasis and invasion. Research indicates that curcumin reduces VEGF and EGFR expression, thereby limiting blood vessel formation (angiogenesis) in CC xenograft models (Fu et al., 2015). Further studies revealed that curcumin and its metabolites, such as tetrahydro curcumin (THC), significantly suppress tumor angiogenesis by inhibiting HIF-1α and VEGF/VEGFR-2 pathways (Fu et al., 2015). Other investigations have shown that methoxy curcumin prevents CC cell migration by inhibiting matrix metalloproteinases (MMP-9, MMP-2) and decreasing the key metastatic protein expression, including GRB2, RhoA, Ras, β-catenin, and N-cadherin, while increasing E-cadherin and NF-κB expression (Liao et al., 2018). Additionally, bisdemethoxycurcumin (BDMC), a structurally similar curcumin derivative, has been reported to inhibit invasion and metastasis by modulating GRB2, RhoA, and uPA expression (Sandur et al., 2007).

Another study by Fei Zheng demonstrated that curcumin’s ability to modulate autophagy and metastasis shows strong potential as a CC treatment. Wound healing experiments further revealed that curcumin and ATG3 silencing inhibited the movement of SiHa and HeLa cells. However, combined, they worked even better, indicating they target similar pathways that control cell migration (Zheng et al., 2024). The study demonstrated that curcumin and ATG3 gene silencing independently elicited a significant reduction in CC cell proliferation, as evidenced by CCK-8 assays, with a synergistic inhibitory effect observed upon combinatorial treatment. Furthermore, both interventions independently attenuated cellular motility, as determined by wound healing assays, with a similar synergistic effect noted upon combination. Concomitant with these phenotypic changes, increased LC3 expression, particularly LC3-II, indicative of enhanced autophagosome formation, was observed, suggesting that autophagy may contribute to the observed anti-neoplastic effects. Molecular docking studies confirmed curcumin’s ability to interact directly with key proteins involved in cell death and proliferation, including MMP2, TGF-β, MMP9, ATG3, P62, and LC3 (Zheng et al., 2024). These interactions indicate curcumin’s role in modulating these proteins’ activity and influencing CC cell behavior.

Despite these promising effects, curcumin’s limited bioavailability, rapid metabolism, and poor systemic retention necessitate alternative delivery strategies. Advances in nanotechnology have been explored to overcome these limitations. A poly (lactic-co-glycolic acid) based curcumin nanoparticle formulation (Nano-CUR) was developed by Mohd S Zaman and his team, wherein they significantly improved curcumin accumulation in CC cells through enhanced endocytosis (Zaman et al., 2016). Nano-CUR demonstrated superior therapeutic effects compared to free curcumin. It effectively reduced cell viability and clonogenicity and was more potent in inducing apoptosis, with late apoptosis rates reaching 63%–88% at higher concentrations. Furthermore, Nano-CUR exhibited enhanced efficacy in arresting CC cells at the G1-S transition phase. Nano-CUR treatment resulted in the most significant tumor growth inhibition among all treatment groups. Additionally, analysis of tumor tissues revealed that both CUR and Nano-CUR suppressed HPV oncoproteins E6 and E7, with Nano-CUR showing greater efficiency in reducing the Ki67 (a proliferation marker) expression (Zaman et al., 2016).

Building upon the example of curcumin, another bioactive compound receiving increasing attention in cervical cancer research is epigallocatechin-3-gallate (EGCG), a major catechin found in green tea. Like curcumin, EGCG exerts pleiotropic anticancer effects through modulation of multiple signaling pathways, and its role in enhancing chemotherapeutic efficacy has been widely documented.

### 3.2 EGCG (epigallocatechin gallate)

Epigallocatechin gallate (EGCG) is a polyphenolic catechin primarily from Camellia sinensis (green tea) leaves. EGCG is the predominant and biologically active catechin constituent of Camellia sinensis, constituting over 40% of the total catechin content, and is recognized for its pleiotropic bioactivities, including antioxidant, anti-inflammatory, antibacterial, and antineoplastic properties. EGCG structurally comprises a flavan-3-ol scaffold bearing multiple hydroxyl substituents, conferring potent free radical scavenging and metal-chelating capabilities. The molecular formula of EGCG is C_22_H_18_O_11_, and its molecular weight is 458.38 g/mol (*Epigallocatechin Gallate* NCBI, 2025). EGCG is a colorless to pale yellow crystalline solid that is highly water-soluble, but its rapid metabolism and low stability under physiological conditions limit its bioavailability. Initially identified for its health benefits due to its presence in green tea, EGCG has gained significant attention for its anticancer effects, particularly in breast, prostate, liver, and CC (Smith et al., 2013).

EGCG exhibits significant anticancer activities through various mechanisms. EGCG halts the cell cycle in CC cells, specifically causing G1-phase cycle arrest in CaSki cells (HPV16-related) and G2/M-phase cycle arrest in HeLa and SiHa cells. This arrest is linked with increased levels of p53, p21, and p27 (tumor suppressor proteins), along with deactivation of EGFR and ERK1/2 (Wang et al., 2023). EGCG attenuates angiogenesis by downregulating vascular endothelial growth factor (VEGF) expression, mediated by inhibiting PI3K/Akt/mTOR pathway and extracellular signal-regulated kinase 1/2 (ERK1/2) signaling pathways. Furthermore, EGCG suppresses the activity of matrix metalloproteinases (MMP-2 or MMP-9), enzymes responsible for extracellular matrix degradation, thereby limiting cancer cell proliferation and invasion (Kciuk et al., 2023).

The study by Panji et al. explored and elucidated the modulatory effects of epigallocatechin gallate (EGCG) and green tea extract on TGF-β-induced epithelial-to-mesenchymal transition (EMT) in CC cells. The research demonstrated that EGCG effectively suppressed the viability of HeLa and SiHa CC cells in a dose-dependent manner. It inhibited TGF-β-induced EMT by downregulating mesenchymal markers like vimentin, ZEB, Slug, Snail, and Twist while upregulating E-cadherin. The mechanism involved reducing ROS levels and inhibiting Smad2/3 phosphorylation, which is crucial in TGF-β-induced EMT and cancer cell migration (Panji et al., 2021). These findings indicated that EGCG could be a potential therapeutic agent to prevent CC progression and metastasis.

Yu Zhu and team designed to determine the impact of EGCG on CC cell growth using various cell lines such as HeLa, CaSki, and SiHa, along with C33A with varying high-risk HPV infections, and to investigate EGCG’s influence on microRNA (miR) expression. It also modulated the expression of key miRNAs involved in CC progression (Zhu et al., 2018). Specifically, EGCG upregulated tumor-suppressive miRNAs like miR-29a and miR-210 while downregulating oncogenic miRNAs like miR-203 and miR-125b in different CC cell lines (Jeong et al., 2024; Zhu et al., 2018). The findings indicate that EGCG’s anticancer effects are partly due to its ability to modify gene expression, suggesting its potential as a treatment for CC.

A recent study by Oladimeji et al. (2020) explored the synergistic anticancer potential of epigallocatechin gallate (EGCG) in the treatment of cervical carcinoma. EGCG, a bioactive flavonoid with potent antioxidant and anticancer properties, was utilized not only for its ability to modulate oxidative stress but also for its influence on key signaling pathways related to cell proliferation and apoptosis. In addition to EGCG, TPP^+^, a delocalized lipophilic cation known for its mitochondrial targeting capacity, was employed to exploit mitochondrial dysfunctions characteristic of cancer cells such as altered membrane potential and disrupted apoptotic signaling. This subcellular targeting strategy enhances drug accumulation in mitochondria, promoting apoptosis through mitochondrial membrane depolarization and caspase activation. Paclitaxel, a widely used chemotherapeutic agent that stabilizes microtubules and induces mitotic arrest, was integrated into the system to exert its cytotoxic effects. Biochemical and imaging analyses in HeLa cells confirmed effective intracellular delivery and mitochondrial localization of the therapeutic agents, resulting in significantly enhanced cytotoxicity compared to the free drug alone. The increased efficacy was attributed to caspase-dependent apoptotic pathways, triggered by both mitochondrial disruption and microtubule stabilization. This study highlights the mechanistically driven combination of antioxidant modulation, mitochondrial targeting, and microtubule interference as a promising strategy for improving the therapeutic index of anticancer agents in cervical cancer therapy​.

A study by Alshatwi et al. (2015) describe the green synthesis of platinum nanoparticles (TPP@Pt) using tea polyphenols as reducing and stabilizing agents, and evaluate their anticancer potential against human cervical cancer (SiHa) cells. The synthesized nanoparticles, characterized as flower-shaped and 30–60 nm in size, demonstrated significant dose- and time-dependent cytotoxicity. Treatment with TPP@Pt induced apoptotic cell death, evident from nuclear fragmentation and chromatin condensation, and caused G2/M phase cell cycle arrest, suggesting DNA damage-mediated growth inhibition. These findings underscore the potential of TPP@Pt as a biocompatible and eco-friendly nanomedicine capable of overcoming limitations associated with conventional platinum-based chemotherapies. These findings collectively highlight the transformative role of nanocarrier systems in optimizing EGCG’s anticancer potential, offering a promising avenue for future clinical translation, particularly in cancers like cervical cancer, where targeted, efficient delivery remains a critical challenge. In addition to curcumin and EGCG, apigenin—a flavonoid abundantly found in various fruits and vegetables—has demonstrated potent anticancer activity in cervical cancer models. Apigenin’s therapeutic relevance stems from its ability to induce apoptosis, inhibit angiogenesis, and suppress metastatic potential through diverse molecular mechanisms.

### 3.3 Apigenin

Apigenin emerged as another bioactive natural compound with promising potential for cervical cancer therapeutics. Apigenin, a naturally occurring flavone found predominantly in Apiaceae and Rutaceae plant families, demonstrates a broad spectrum of bioactivities, including antioxidant, anti-inflammatory, neuroprotective, and antineoplastic properties. Its chemical structure, characterized by a flavone scaffold with hydroxyl substituents at positions 4, 5, and 7, confers potent free radical scavenging capabilities. Apigenin, with the molecular formula C_15_H_10_O_5_ and a molecular weight of 270.24 g/mol, presents as a yellow crystalline solid with limited aqueous solubility, which influences its systemic absorption and bioavailability (*Apigenin |C15H10O5 | CID 5280443 -* NLM, 2025a). Apigenin was first recognized for its medicinal properties due to its presence in traditional herbal remedies, and it has been widely studied for its anticancer potential in cervical, breast, lung, prostate, and colon cancers. Recent research highlights apigenin’s ability to modulate key signaling pathways, induce apoptosis, and suppress tumor progression, making it a good candidate for cancer prevention and therapy (Rahmani et al., 2022).

A study conducted by Zhang et al. (2020) explored the anticancer potential of apigenin in cervical cancer, focusing particularly on its role as a phytoestrogen and modulator of estrogen receptor (ER) signaling. They had used histamine, a biogenic amine, a promoter for tumor growth in hormone-dependent cancers by altering ER expression. In cervical cancer specifically, they found that histamine stimulated cell proliferation both *in vitro* and *in vivo*, mainly by upregulating ERα and downregulating ERβ, leading to an imbalanced ER signaling environment conducive to tumor progression. To investigate whether apigenin could counteract this effect, they worked on HeLa cervical cancer cells and tumor-bearing mice with apigenin. Their findings showed that apigenin inhibited HeLa cell proliferation in a dose-dependent manner. *In vivo*, apigenin significantly reduced tumor volume and weight in xenograft models. Mechanistic studies revealed that apigenin reversed the histamine-induced alterations in ER expression by downregulating ERα and upregulating ERβ, effectively restoring the ERβ/ERα ratio. This shift in receptor expression attenuated the pro-growth signaling induced by histamine. Further analyses demonstrated that apigenin’s modulation of ER signaling suppressed the activation of the PI3K/Akt/mTOR pathway—a key regulator of cancer cell survival, proliferation, and resistance to apoptosis. The study also confirmed that apigenin enhanced the expression of pro-apoptotic markers, such as Bax, and reduced the levels of angiogenesis-promoting factors like VEGF in serum. Interestingly, apigenin also restored serum estradiol (E2) levels reduced by histamine, suggesting its potential to stabilize endocrine function through competitive binding to ERα.

In addition to its role in apoptosis, apigenin is crucial in inhibiting metastasis. It has been shown to prevent cancer cells from migrating and invading by decreasing the production of MMP-2/MMP-9, which is needed to degrade the extracellular matrix. By interfering with the PI3K/Akt signaling pathway, apigenin reduces the metastatic potential of cancer cells, as demonstrated in melanoma and breast cancer models. Furthermore, apigenin modulates key signaling pathways involved in tumor progression. It inhibits the PI3K/Akt pathway, decreasing the proliferation of cells and survival, and suppresses the MAPK/ERK pathway, reducing tumor growth. It also downregulates NF-κB activation, which is crucial for inflammation-associated tumor progression, and modulates the Wnt/β-catenin pathway, thereby reducing cancer stem cell properties and tumorigenesis (Rahmani et al., 2022; Yan et al., 2017).

Li et al. (2024) investigated the role of apigenin 7-glucoside (A7G) in suppressing hypoxia-induced malignant phenotypes in cervical cancer cells. Under hypoxic conditions, HeLa cells exhibited increased proliferation, migration, invasion, stemness, and resistance to chemotherapy, reflecting a more aggressive cancer profile. Treatment with A7G significantly reversed these effects in a dose-dependent manner by enhancing chemosensitivity and reducing tumorigenic behaviors. Mechanistically, A7G promoted the activation and nuclear localization of the tumor suppressor p16 by interacting with anion exchanger 1 (AE1), which under hypoxia otherwise sequesters p16 in the cytoplasm. Silencing of p16 abolished the therapeutic effects of A7G, confirming that its anticancer action is p16-dependent. This study highlights A7G as a potential therapeutic agent for cervical cancer, particularly effective in targeting hypoxia-driven tumor progression.

The study by Souza et al. (2017) revealed the differential cytotoxic effects of apigenin on a panel of human CC cell lines, including HeLa, CaSki, SiHa, and C33A, in comparison to a non-tumorigenic epithelial cell line, HaCaT. The results demonstrated that apigenin selectively induced apoptosis in the carcinoma cell lines while exhibiting minimal cytotoxicity toward the normal epithelial cells. This apoptotic effect was attributed to mitochondrial oxidative stress, as apigenin increased ROS levels, hydrogen peroxide (H_2_O_2_), and lipid peroxidation while decreasing mitochondrial membrane potential. Furthermore, the study highlighted apigenin’s ability to inhibit cancer cell migration and invasion, signifying its potential as a promising candidate for CC therapy.

**FIGURE 3:**
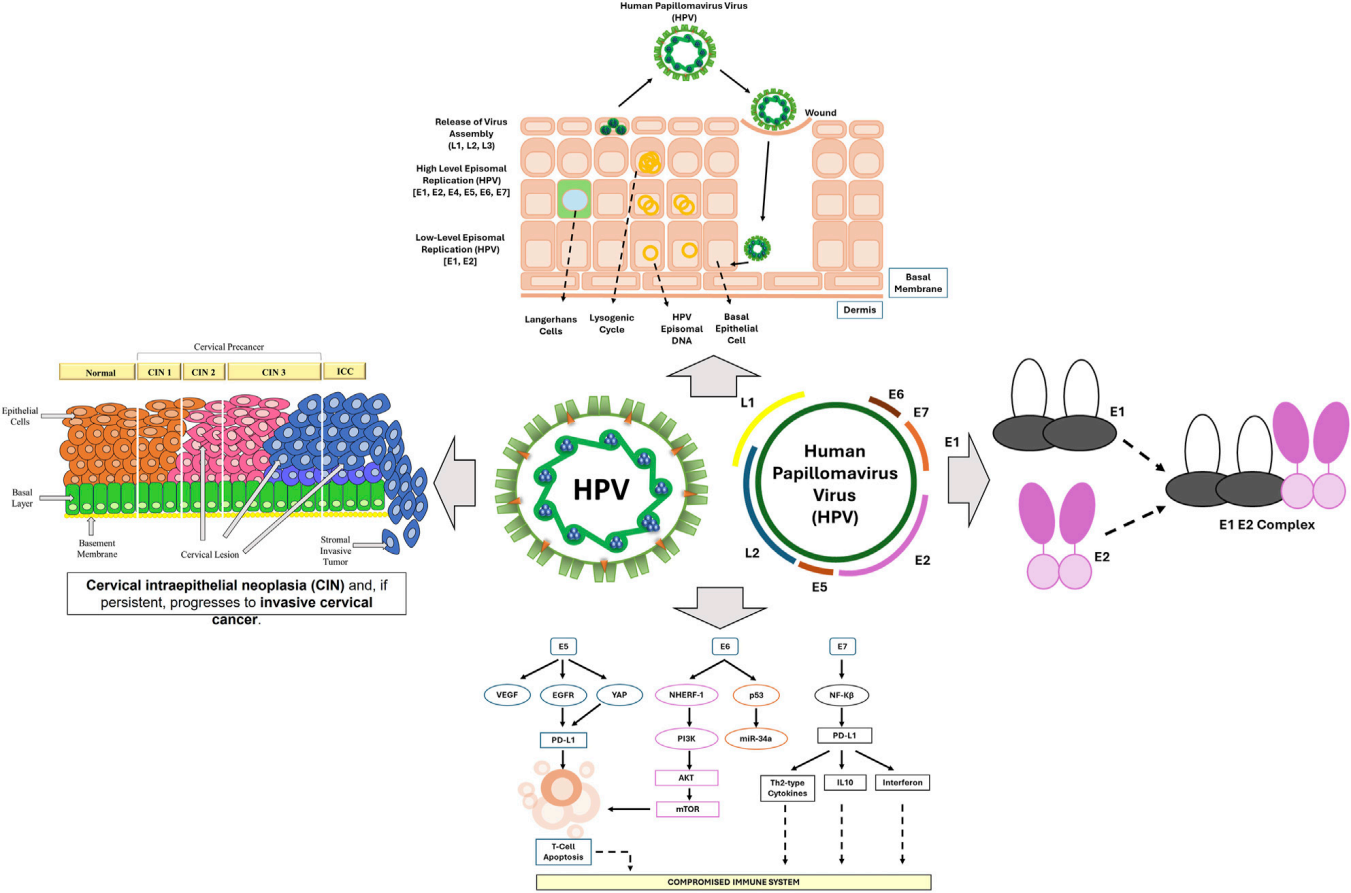
Inhibitory effects of curcumin, EGCG, and apigenin on key signaling pathways involved in cervical cancer. Red arrows indicate suppression of specific molecular interactions, highlighting the therapeutic potential of these compounds in cervical cancer treatment.

Another significant study by Chen et al. (2022) elucidated the molecular mechanisms underlying the antineoplastic effects of apigenin in CC, utilizing both *in vivo* and *in vitro* models. Apigenin-induced G2/M cell cycle arrest and mitochondrial-mediated apoptosis in HeLa and C33A cells through the downregulation of focal adhesion kinase (FAK) signaling, encompassing FAK, integrin β1, and paxillin as well as the inhibition of the PI3K/AKT/mTOR signaling pathway. In a cervical tumor xenograft mouse model, apigenin significantly attenuated tumor growth, reinforcing its potential as a chemotherapeutic agent. Through the modulation of key molecular pathways implicated in oncogenesis, apigenin demonstrated its capacity to induce apoptosis, impede cellular migration, and suppress tumor growth, underscoring its therapeutic promise for CC management (Figure 3).

A study by Bonilla-Vidal et al. (2023) investigated a nanostructured lipid carrier (NLC) system to improve the therapeutic profile of apigenin (APG), a flavonoid with well-documented anticancer properties but poor water solubility and bioavailability. The NLCs were designed to encapsulate APG along with rosehip oil, a natural component known for its antioxidant and antitumoral activities, creating a dual-function system where both the active compound and the lipid matrix contribute to anticancer efficacy. The optimized APG-NLCs demonstrated high encapsulation efficiency, colloidal stability, and a sustained release profile characterized by an initial burst followed by prolonged drug release. These properties supported a controlled delivery of APG, enhancing its cellular availability and therapeutic potential. Biological evaluations confirmed that the APG-NLCs selectively inhibited proliferation of several cancer cell lines, including leukemia (MV4-11), lung carcinoma (A549), and triple-negative breast cancer (MDA-MB-468), while exhibiting minimal cytotoxicity toward non-tumorigenic breast epithelial cells (MCF-10A). Importantly, the formulation also displayed marked antiangiogenic activity in the chorioallantoic membrane (CAM) assay, indicating its potential to interfere with tumor vascularization, an essential mechanism in cancer progression. Flow cytometry analyses also showed that the nanocarriers were efficiently internalized by cancer cells, supporting their capacity to enhance intracellular delivery of APG. These findings underline the role of NLCs not only as passive carriers but also as bioactive components due to the presence of rosehip oil, which may exert synergistic effects with APG (Table 1).

**TABLE 1:** Comparative analysis of the physicochemical traits, anticancer mechanisms, molecular targets, and synergistic potential of Curcumin, EGCG, and Apigenin, highlighting their relevance for nanocarrier-based theranostic applications.

Parameter	Curcumin	EGCG (epigallocatechin gallate)	Apigenin
Natural Source	Curcuma longa (turmeric)	Green tea (Camellia sinensis)	Parsley, chamomile, celery, and other medicinal herbs
Chemical Formula/Weight	C_21_H_20_O_6_/368.38 g/mol	C_22_H_18_O_11_/458.38 g/mol	C_15_H_10_O_5_/270.24 g/mol
Physicochemical Properties	Yellow crystalline compound; low water solubility	Slightly soluble in water; greenish color; potent antioxidant	Yellow crystalline compound; moderate water solubility
Primary Anticancer Mechanisms	Induces apoptosis via ROS, DNA damage, mitochondrial dysfunction	Induces apoptosis, autophagy, and DNA fragmentation via mitochondrial ROS generation	Promotes cell cycle arrest and apoptosis; inhibits migration and invasion
Cellular Pathways Targeted	Modulates p53, NF-κB, PI3K/Akt, and JAK/STAT pathways	Downregulates EGFR, PI3K/AKT, and MAPK pathways	Suppresses Wnt/β-catenin, PI3K/AKT, MAPK, NF-κB, and JAK/STAT3 pathways
Effect on Cell Cycle	Induces G2/M phase arrest	Induces G1 phase arrest	Induces G2/M phase arrest
Apoptotic Effects	Upregulates Bax, p53, cytochrome-c; downregulates Bcl-2	Upregulates Bax, caspase-3; downregulates Bcl-2, cyclin D1	Activates caspase-3/9, increases Bax/Bcl-2 ratio
Anti-inflammatory Effects	Inhibits TNF-α, IL-6, and NF-κB	Reduces inflammation via cytokine suppression	Inhibits COX-2, IL-6, and NF-κB
Anti-metastatic/Anti-angiogenic	Reduces migration, invasion, angiogenesis	Inhibits tumor growth and blood vessel formation	Inhibits VEGF, MMPs; suppresses EMT
Challenges	Low bioavailability due to poor absorption	Poor stability and low systemic availability	Limited bioavailability and rapid metabolism
Synergistic Potential	Enhances effects when combined with chemotherapeutics (e.g., cisplatin)	Shows synergistic activity with paclitaxel	Potentiates doxorubicin and other drugs

## 4 Bioavailability and challenges in clinical translation

Despite their promising therapeutic potential, natural bioactive compounds face significant challenges in clinical translation due to their inherent limitations, including poor bioavailability, low stability, and rapid metabolism. Many bioactive compounds, such as curcumin, EGCG, and apigenin, suffer from poor aqueous solubility, leading to limited absorption and bioavailability in physiological conditions. Their extensive first-pass metabolism and rapid clearance further restrict their systemic exposure and therapeutic efficacy (Luo et al., 2024). Additionally, these compounds often have low chemical stability, undergoing degradation due to oxidation, pH changes, or enzymatic hydrolysis before reaching their target sites. Such challenges hinder their clinical application, necessitating innovative strategies to enhance their bioavailability and therapeutic potential.

Various approaches have been explored to overcome these limitations, including nanocarrier-based delivery systems, synthetic derivatives, and combination therapies. Nanocarriers such as liposomes, solid lipid nanoparticles, polymeric nanoparticles, and dendrimers have been developed to improve bioactive compounds’ solubility, stability, and cellular uptake (Din et al., 2017). For example, curcumin-loaded polymeric nanoparticles have demonstrated enhanced bioavailability and anticancer efficacy in CC models. At the same time, EGCG encapsulated in liposomal formulations has shown improved stability and sustained release properties (Nasery et al., 2020). Another promising approach involves the development of synthetic derivatives and analogs with improved pharmacokinetic properties. Structural modifications, such as conjugation with hydrophilic moieties or incorporation into prodrug forms, have been explored to enhance these compounds’ solubility and metabolic stability. Additionally, combination therapy strategies integrating bioactive compounds with conventional chemotherapeutic agents have been investigated to improve treatment outcomes. Curcumin, for instance, has been shown to sensitize CC cells to cisplatin and radiation therapy by modulating key signaling pathways such as NF-κB and PI3K/Akt, thereby enhancing the efficacy of standard treatments (Mokhtari et al., 2017).

**FIGURE 4:**
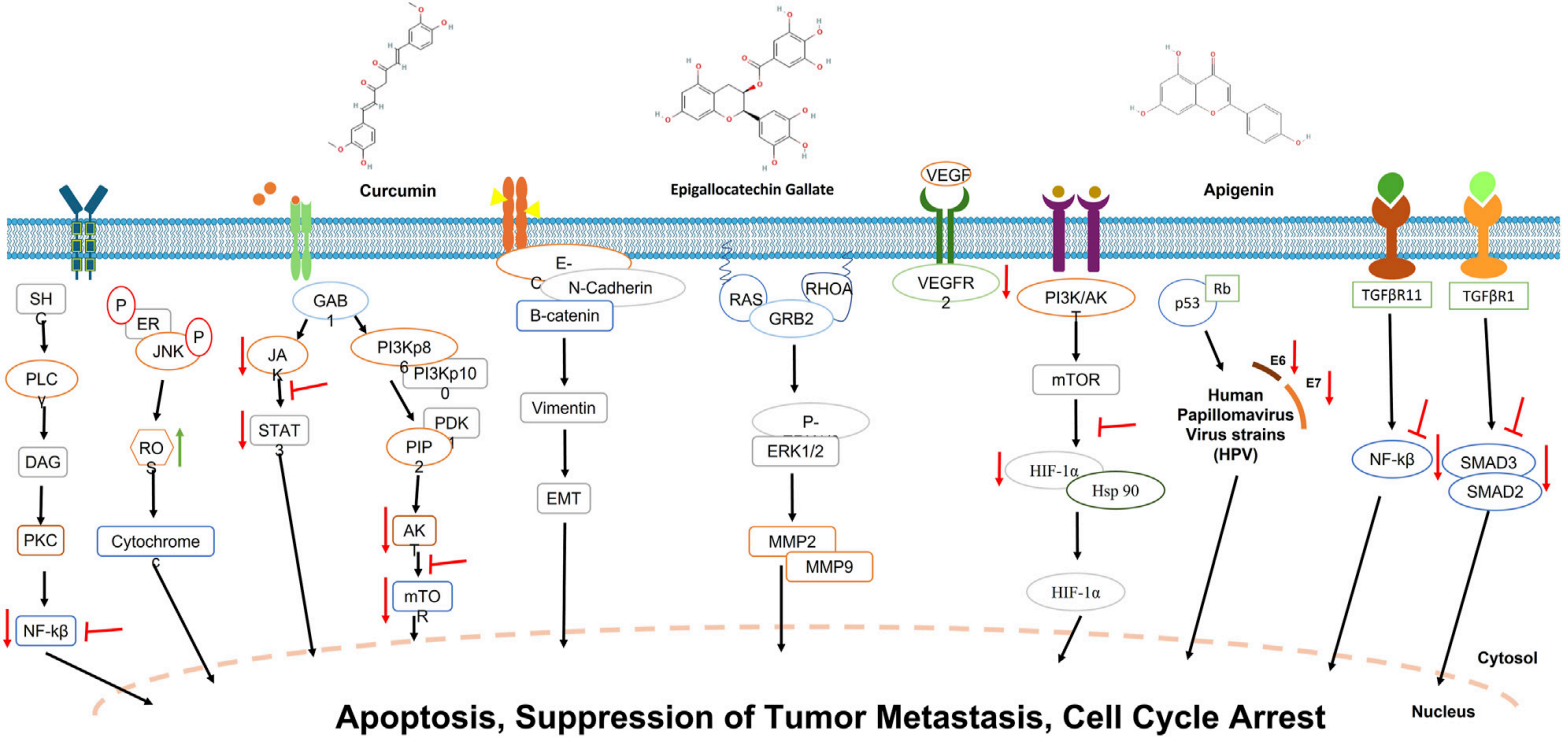
This schematic highlights the diagnostic and therapeutic roles of nano-formulated Curcumin, EGCG, and Apigenin in cancer theranostics.

Bioactive compounds demonstrate considerable promise in theranostic applications for cervical cancer, offering the dual benefit of therapeutic efficacy and diagnostic capability within a single platform. These compounds modulate key oncogenic pathways, such as PI3K/AKT/mTOR, NF-κB, and STAT3, to suppress tumor growth, induce apoptosis, and inhibit metastasis. Curcumin, for instance, targets HPV oncoproteins (E6/E7) and enhances chemosensitivity, while EGCG suppresses angiogenesis and immune evasion pathways. Apigenin promotes cell cycle arrest and synergizes with conventional therapies to overcome drug resistance. Nanotechnology further enhances their efficacy by improving bioavailability and enabling targeted delivery—curcumin-loaded liposomes (Pourhanifeh et al., 2020), EGCG-conjugated gold nanoparticles (Surendran et al., 2018), and apigenin-encapsulated PLGA nanoparticles (Surendran et al., 2018) have demonstrated improved tumor penetration and real-time imaging capabilities in cervical cancer models. Additionally, these compounds exhibit dual diagnostic utility by serving as fluorescence probes for tumor detection and as adjuvants to enhance contrast in imaging modalities like MRI and PET. Their multi-target mechanisms and compatibility with nano-formulations position them as promising candidates for precision theranostics in cervical cancer management (Figure 4).

Despite these advancements, the clinical translation of bioactive compounds remains challenging due to issues related to large-scale production, regulatory approval, and inter-individual variability in drug metabolism. Future research should focus on optimizing formulation strategies, conducting well-designed clinical trials, and elucidating precise mechanisms of action to facilitate the integration of bioactive compounds into mainstream clinical practice. Addressing these challenges requires harnessing the full therapeutic potential of plant-derived bioactive compounds in CC treatment. As known, phase 2 trials for EGCG have demonstrated its potential in reducing HPV-induced lesions, yet curcumin’s early-phase trials underscore the need for improved formulations to achieve therapeutic doses (Garcia et al., 2014; ClinicalTrials.gov, 2023). While promising in preclinical models, Apigenin requires further clinical validation to establish its efficacy and safety profile. Moreover, ongoing and completed clinical trials have provided valuable insights into the therapeutic potential of these bioactive compounds in CC treatment. Several studies have investigated the effectiveness of curcumin as an adjuvant therapy, demonstrating its ability to enhance the response to chemotherapy and radiotherapy while minimizing adverse effects. Similarly, EGCG has been explored for its role in sensitizing CC cells to radiation and chemotherapy, improving treatment outcomes. Although less studied in clinical settings, Apigenin has shown promise in preclinical models, warranting further investigation in human trials (Table 2).

**TABLE 2:** Overview of clinical trials investigating the therapeutic potential of epigallocatechin gallate (EGCG) and curcumin in cervical cancer and HPV-associated lesions.

Clinical trial no.	Phase	Targeted drug	Title of study	References
NCT05625308	Not Applicable	Epigallocatechin gallate (EGCG)	Effect of Natural Compounds on the Severity of HPV-induced Cervical Lesions	ClinicalTrials.gov (2022)
NCT06314113	Not Applicable	Epigallocatechin gallate (EGCG)	Evaluation of Oral EGCG Treatment for L-SIL Associated With HPV Infection (EGCG-HPV)	ClinicalTrials.gov (2024c)
NCT06098456	Phase 2	Epigallocatechin gallate (EGCG)	Epigallocatechin Gallate and Other Antural Compounds in HPV Infections (EGCGHPV)	ClinicalTrials.gov (2024b)
NCT00303823	Phase 2	Epigallocatechin gallate (EGCG)	Green Tea Extract in Preventing CC in Patients With Human Papillomavirus and Low-Grade Cervical Intraepithelial Neoplasia	ClinicalTrials.gov (2015)
NCT02554344	Early Phase 1	Curcumin	Effect of Curcumin in Treatment of Squamous Cervical Intraepithelial Neoplasias (CINs)	ClinicalTrials.gov (2016)
NCT06080841	Not Applicable	Curcumin	Curcumin Supplementation in CC	ClinicalTrials.gov (2023)
NCT05947513	Not Applicable	Curcumin	Concomitant Curcumin Palliative Radiotherapy in Advanced CC Trial (CuPRAC)	ClinicalTrials.gov (2024a)
NCT04266275	Phase 2	Curcumin	Topical Curcumin for HPV-Related Cervical Disease	ClinicalTrials (2025)
NCT03192059	Phase 2	Curcumin (Supplementation)	Study of Pembrolizumab, Radiation and Immune Modulatory Cocktail in Cervical/Uterine Cancer (PRIMMO)	Study of Pembrolizumab (2021)

## 5 Discussion

CC remains one of the global health burdens, particularly in areas with limited access to early detection and standard treatment strategies. While conventional therapies such as chemotherapy, surgery, and radiotherapy offer proven benefits, these are often offset by debilitating side effects, treatment resistance, and the possibility of recurrence. With the growing understanding of HPV-driven cervical carcinogenesis, there is an increasing focus on alternative therapeutic strategies, including plant-derived bioactive compounds such as curcumin, epigallocatechin gallate (EGCG), and apigenin. These compounds have demonstrated promising anticancer effects by targeting multiple molecular pathways, including PI3K/Akt, NF-κB, MAPK/ERK, and Wnt/β-catenin, while also playing crucial roles in inducing apoptosis, inhibiting proliferation, suppressing metastasis, and modulating immune responses. Furthermore, their ability to suppress HPV oncoprotein expression highlights their potential in cancer prevention and therapy.

Despite competing for preclinical evidence, the clinical practice of these bioactive compounds faces various challenges due to their poor bioavailability, rapid metabolism, and limited systemic distribution. Nanotechnology-based formulations, synthetic derivatives, and combination therapies have been explored to enhance their pharmacokinetic properties and therapeutic efficacy. Nanoparticle-based drug delivery systems, liposomal formulations, and polymeric carriers have demonstrated improved solubility, stability, and cellular uptake, potentially overcoming these limitations. Additionally, combining bioactive compounds with conventional chemotherapeutic agents has shown promise in enhancing treatment sensitivity, reducing toxicity, and improving patient outcomes.

Emerging clinical trials have begun to validate the efficacy of these bioactive compounds in adjuvant and combination therapies. Curcumin has been explored as a radiosensitizer and chemosensitizer; EGCG has demonstrated potential in inhibiting angiogenesis and metastasis, while apigenin has shown promise in targeting cell cycle regulation and apoptosis. However, more well-structured, large-scale clinical trials are required to establish their long-term safety, efficacy, and optimal dosing regimens.

Beyond direct anticancer effects, natural products may hold broader implications in modulating inflammation, immune responses, and even microbiome interactions. The relationship between the gut microbiota, inflammation, and CC progression presents a potential avenue for future research, especially as certain bioactive compounds, such as berberine, have demonstrated immunomodulatory and microbiome-altering properties. Further interdisciplinary studies integrating pharmacokinetics, pharmacodynamics, metabolomics, and microbiome research could pave the way for more effective, personalized therapeutic strategies.

However, challenges remain in optimizing drug delivery, ensuring safety, and overcoming drug resistance. The low absorption, extensive metabolism, and rapid elimination of bioactive compounds require advanced formulation techniques. Additionally, combination therapies with conventional drugs must be carefully evaluated to avoid adverse interactions. One potential strategy is incorporating these compounds into dietary interventions, leveraging their natural occurrence in fruits, vegetables, and herbal products. While dietary supplementation may enhance preventive benefits, its therapeutic effectiveness remains limited, necessitating further investigation.

Plant-derived bioactive compounds show significant advantages as a complementary strategy for CC, offering potential benefits in treatment, prevention, and improved therapeutic outcomes. Their ability to overcome various cancer pathways with low toxicity makes them ideal for integrative cancer care usage. While bioavailability and clinical validation challenges persist, continued research on innovative delivery systems, mechanistic insights, and clinical applications will be crucial for their successful translation into effective, low-toxicity therapeutic options. A multidisciplinary approach involving cancer researchers, chemists, and clinical experts is necessary to unlock the full potential of natural compounds in combating CC.
